# Multiresidue Determination of 26 Quinolones in Poultry Feathers Using UPLC-MS/MS and Their Application in Residue Monitoring

**DOI:** 10.3390/molecules28093738

**Published:** 2023-04-26

**Authors:** Zhanteng Song, Zhiming Xiao, Xia Fan, Hongting Zhuang, Yang Li, Jingrong Zhu, Duoyong Zhao, Maerhaba Paerhati, Decheng Suo

**Affiliations:** 1 Institute of Quality Standards and Testing Technology for Agricultural Products, Xinjiang Academy of Agricultural Science, Urumqi 830091, China; 18293897471@163.com (Z.S.); zhujr2030@163.com (J.Z.); luckydyz@163.com (D.Z.); marhaba524@126.com (M.P.); 2 Institute of Quality Standards and Testing Technology for Agricultural Products, Chinese Academy of Agricultural Science, Beijing 100081, China; 3 Liaoning Agricultural Development Service Center, Shenyang 110000, China

**Keywords:** poultry feathers, quinolones, UPLC-MS/MS, multi-absorption purification, simultaneous measurement

## Abstract

As a non-traditional sample matrix, feather samples can be used to effectively monitor antibiotic addition and organismal residue levels in poultry feeding. Therefore, an ultra-performance liquid chromatography–tandem mass spectrometry (UPLC-MS/MS) method was developed to simultaneously determine the residue levels of 26 quinolones in poultry feathers. The feather samples were extracted by sonication with a 1% formic acid and acetonitrile mixture in a water bath at 50 °C for 30 min, purified by the adsorption of multiple matrix impurities, dried with nitrogen, redissolved, and analyzed by UPLC-MS/MS. The linearity, limit of detection (LOD), limit of quantification (LOQ), recovery and precision were calculated. The 26 antibiotics demonstrated good linearity in the linear range. The recoveries and coefficients of variation were 78.9–110% and <13.7% at standard spiked levels of 10, 100 and 200 μg/kg, respectively. The LOD and LOQ were 0.12–1.31 and 0.96–2.60 μg/kg, respectively. The method also successfully identified quinolone residues in 50 poultry feather samples. The results showed that quinolones can accumulate and stabilize for a certain period of time after transferring from the body to the feathers of poultry.

## 1. Introduction

Quinolones (QNs) are a class of synthetic antibiotics that has developed rapidly. Their compounds contain pyridine keto acid (1,4-dihydro-4-oxypyridine-3-carboxylic acid) as a basic structure with carbonyl, carboxyl and other groups [1]. This class of drug promotes the growth of animals, reduces costs because it prevents and treats the diseases of livestock and aquatic animals, and is widely used in aquaculture as a veterinary medicine and feed additive [2]. However, the massive use of quinolones has resulted in the accumulation of residues in poultry products, which are recycled to the human body and cause various hazards. An excessive intake of quinolones mainly manifests in direct toxic reactions (nausea, vomiting, vertigo, palpitations, and cardiovascular system problems), allergic reactions (skin damage and toxicity due to photodegradation products). Bacterial drug resistance to quinolones has also increased and attracted increasing attention [3]. Molecular mechanisms of fluoroquinolone resistance were found in a clinical isolate of Escherichia coli that was highly susceptible to nalidixic acid but resistant to fluoroquinolones. China’s Ministry of Agriculture issued Announcement No. 2292, which stipulates that the addition and use of four veterinary drugs is prohibited as of 31 December 2016 [4]. Since 2005, the U.S. Food and Drug Administration (FDA) has banned the use of enrofloxacin for the treatment of bacterial infections in poultry as scientific evidence suggests that Campylobacter-resistant species have emerged in chickens and turkeys treated with antimicrobials [5]. The current detection methods for quinolone residues in livestock and poultry products are mainly focused on the muscles, liver and feces [6,7,8,9]. In poultry, after the body receives antibiotic treatment, the drug residence time varies in different body parts due to metabolism, and the residual level in tissues immediately falls below the measurable limit; hence, the timeliness of this monitoring method is limited [10]. This phenomenon is known as the paradoxical effect of quinolones: high concentrations of quinolones are less bactericidal. Moreover, the excretion rate of live animals is high, and the detection window of feces is short. Antibiotic residues can only be detected during dosing and for a few days after slaughter [11]. Feathers are often used as a source of waste or cheap protein feed in poultry breeding. The content of residual antibiotics detected in feather powder is higher than that of the conventional matrix [12]. Thus, feathers have been proposed as a substitute sample material for detecting antibiotic residues in a long time window, with the advantages of a stable drug presence, non-invasive nature, convenient transportation, and preservation [13]. Antibiotic residue surveillance is also important and includes early warning functions for the detection of public health events. Increasing needs demand the development of rapid and sensitive techniques for the detection of various antibiotics in trace amounts. Feathers could be an effective target tissue for antibiotic residue detection for an early warning against the presence of antibiotics. However, only a few studies have examined antibiotic residues using poultry feathers as a substrate. Feathers can be used to detect residues of antimicrobial agents because the drug may bioaccumulate in the internal structures of the feathers [14]. The combined structure, the feather primordium, then grows into a filament within a follicle, branches internally, and produces a down feather by hatching. A feather has a hollow tube sac structure, and drugs enter the feather shaft and feather branch through the metabolic power path. The drugs accumulating in feathers are not affected by other tissues, are stable, and can be detected even for a longer time limit when compared with drugs in other tissues of the body [15]. At present, the commonly used detection methods for quinolone residues are high-performance liquid chromatography (HPLC) [16,17,18], an enzyme-linked immunosorbent assay (ELISA) [19,20,21], and UPLC-MS/MS [22,23,24]. ELISA uses highly specific reactions of an antigen and antibody under the catalysis of an enzyme to carry out detection and analysis and has the advantages of good selectivity, strong specificity, and high sensitivity. HPLC is widely used to detect quinolone residues, but some compounds with good thermal stability have a relative shortage of separation and confirmation. UPLC-MS/MS combines the separation function of chromatography with the qualitative function of mass spectrometry. It can realize the qualitative and quantitative analyses of complex mixtures, simplify the pretreatment of samples, and retain the advantages of high selectivity and high sensitivity. UPLC-MS/MS has unique benefits for the separation and identification of compounds with a high boiling point, thermal instability, and poor volatility. These properties make UPLC-MS/MS an important method for quinolone residue detection. Relevant studies have been reported on the analysis of quinolone antibiotic residues in poultry feathers [25,26,27]. D.C. Love et.al. analyzed feather meal (*n* = 12 samples) for ciprofloxacin, enrofloxacin, norfloxacin, and ofloxacin, using EPA method 1694 and employing liquid chromatography–tandem mass spectrometry (LC-MS/MS) [14]. Larissa J.M. Jansen established a method for the analysis of six QNs in chicken feathers [28]. Apart from these examples, there is little information on the identification of quinolone drug residues in poultry feathers. The overall objective of this study was to establish a simple and effective method for the analysis of quinolone antibiotic residues in poultry feathers as a means of determining the approximate level of drug use in poultry during feeding.

## 2. Results and Discussion

### 2.1. Chromatographic Condition Optimization

**Figure 1:**
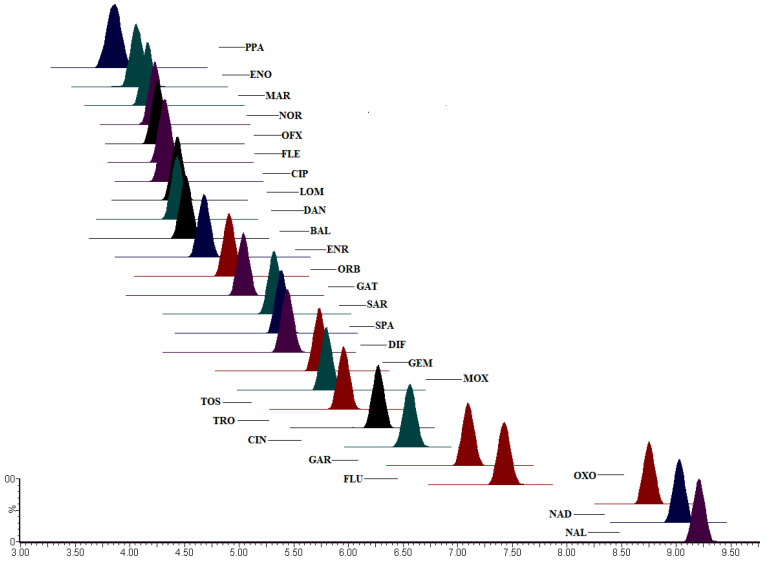
The mixed standard solutions of 26 QNs were injected by a mobile phase of 0.1% FA–water and ACN gradient elution under the adjustment of the instrument to the optimal condition. The ion flow chromatograms of the 26 compounds were obtained by injecting samples when the parameters reached equilibrium. Under this instrument condition, perfect separation was achieved with sharp peaks and no tailing; therefore, it was determined to be the best condition for the detection of the 26 QNs residues by the instrument. All compounds are listed in order of peak appearance time. Typical MRM chromatograms of 26 quinolones.

Quinolone contains carboxyl and piperazine nitrogenous bases in its molecular structure and is cationic in an acidic solution. [M + H]^−^ excimer ions are easily formed in the positive ion mode of the electrospray. Therefore, ESI and multi-stage reaction monitoring mode (MRM), which effectively reduce matrix interference and improve the sensitivity of the method, were used for scanning. According to the acid–base amphoteric chemical properties of the quinolones, their dissociation state changes with pH, and the pH of the mobile phase will have a significant impact on the separation and retention of the quinolones in chromatographic columns. Adding an appropriate amount of formic acid into the mobile phase could improve the ionization efficiency of analytes in the positive ion mode of the electrospray mass spectrometry and effectively improve the peak shape. The number of drugs to be detected was large; hence, isocratic elution could not effectively separate these drugs, methanol had a high viscosity, and the column equilibrium time might be longer during gradient elution. Therefore, by comparing the effects of formic acid/water-acetonitrile with different volume fractions on each analyte, 0.1% formic acid/water-acetonitrile was finally selected as the mobile phase. Finally, the ACQUITY UPLC BEH C18 (150 × 3.0 mm, 1.7 μm) and ACN + 0.1% formic acid were selected as the optimum column and mobile phase, respectively, because they provide narrow peaks, high sensitivity, and an optimal resolution for each analyte, ranging from 3.82 min (pipemidic acid) to 8.99 min (nalidixic acid). The chromatograms are shown in Figure 1.

### 2.2. Extraction Optimization

**Figure 2:**
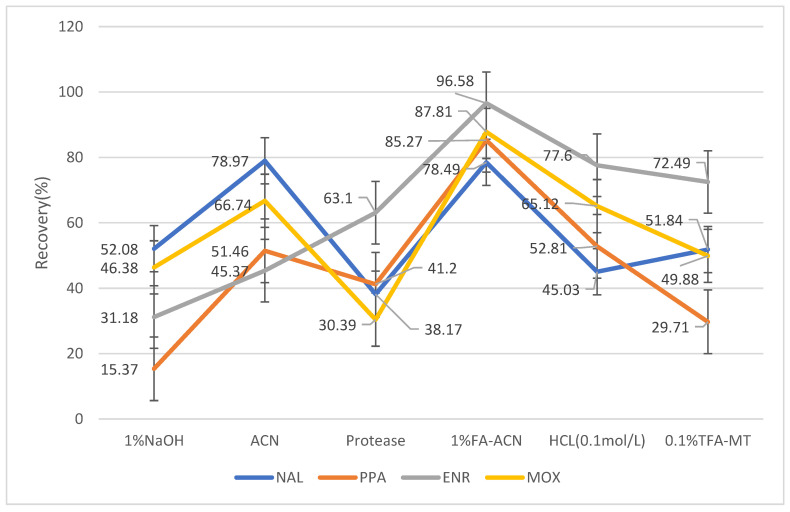
NAL, PPA, ENR, and MOX as representatives of different generations of development in the research history of QNs, respectively. By examining the recoveries of the samples, the recoveries of the four compounds at 1% FA-ACN reached 78.49%, 85.27%, 96.58%, and 87.81% after treatment with different extracts, respectively. The highest recovery was achieved compared to other extracts. Therefore, it was decided to use 1% FA-ACN as the extraction solution for the method. Recovery efficiency of QNs by different extraction methods.

Various solutions have been used for the extraction of target components, according to different materials in several quinolone detection and analysis reports. It is mainly attributed to acid extraction, alkali extraction, organic solution extraction, and mixed solution extraction [29,30]. However, only a few studies have reported the extraction of quinolones from feathers. According to other residual component analysis reports based on feathers [23,31,32,33,34], there are also differences in the results achieved by different extraction methods. In this study, different extraction methods were selected to study the extraction efficiency of QNs in feathers in which nalidixic acid, piperidic acid, enrofloxacin, and moxifloxacin were used as representatives of different generations of drug development of QNs, respectively. The extraction recovery efficiency of the HCL solution (0.1 mol/L), NaOH solution (1 mol/L), acetonitrile (chromatographically pure), protease solution (10 g/L), 0.1% TFA–methanol solution, and the 1% formic acid and acetonitrile mixture were compared for the four generations of QNs. Among them, acid, alkali solution, and protease solution extraction was performed by shaking bed vibration, and organic solvent extraction was performed by ultrasonic extraction with an enzymatic treatment time of no less than 18 h. The extraction efficiencies of the different extraction methods for each compound are shown in Figure 2.

The crude protein component in the chemical composition of feathers was as high as 85%; therefore, hydrolyzing the feathers with a protease solution was considered. However, feather protein is an insoluble protein, and several disulfide bonds with a cable structure, which are formed between the peptide chains inside the protein skeleton, hinder the function of proteolytic enzymes. Common proteases (e.g., trypsin, pepsin, and papain) have difficulty in breaking down feather samples effectively [35]. Enzymatic hydrolysis usually requires hot pressing, which is not conducive to the extraction and detection of target drugs. Acetonitrile can precipitate protein with less interference from impurities, but the coagulation of protein is not conducive to the effective extraction of target substance, resulting in relatively low recovery rate, and quinolones are more stable in a neutral acid environment [36]. Moreover, the enzymatic extraction method was excluded as it is time-consuming and complicated. The recovery rate of the 0.1% TFA methanol solution in the third and fourth generations of quinolone antibiotics was low. The HCl and NaOH solutions were extracted and purified through a solid-phase extraction column to reduce the adverse effect of acid-base extraction on UPLC. However, generations 3 and 4 of quinolone demonstrated no good retention, resulting in a strong ion-inhibition effect and affecting the sensitivity and accuracy of the quinolone. At the same time, the purification process via extraction column is relatively complicated and time-consuming [37,38]. Based on the recovery rates of several extracts, 1% formic acid-acetonitrile was finally chosen as the extraction solution for quinolones in poultry feathers.

### 2.3. Selection of Purification Methods

The unpurified extract of acidified acetonitrile will lead to a strong matrix effect. Multiple mechanisms of impurity adsorption purification were selected to remove the interference of the matrix after the extraction of acidified acetonitrile to purify and better remove impurities from the extract and to solve the problem of the lengthy analysis time of the column-passing treatment [39]. Multifunction adsorption purification is a newly proposed method based on medium-dispersion solid-phase extraction [40]. Through a variety of functional adsorption materials, the main interfering impurities in the sample are adsorbed to effectively remove phospholipids, fats, and some proteins that may exist in the matrix, whereas the tested substances are left in the sample solution to achieve the purpose of purification and enrichment [24].

**Figure 3:**
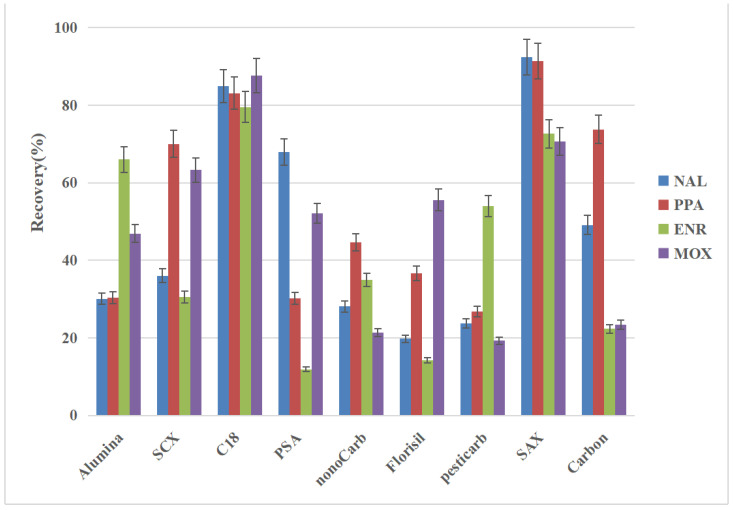
The recovery efficiencies of the nine adsorbent materials for the four QNs are shown in the figure. The adsorption and purification effect of different materials varies. Among them, C_18_ and SAX showed high and balanced purification efficiency for the four QNs. Other materials have a large difference in efficiency after purification. Therefore, C_18_ and SAX were selected as backup adsorption and purification materials. Recovery rate of quinolone drugs by different adsorption materials.

Nine adsorbent materials (Table 1) were selected for adsorption purification experiments. The specific experimental steps were as follows. Blank feather samples were obtained. In a 50 mL centrifuge tube, 1 g was accurately weighed, and the appropriate amount of QNs mixed in a standard solution and its internal standard solution was added to create content of 10 mg/kg relative to that in the tissue, which was vortex-mixed well. Then, 30 mL of acetonitrile extraction solution of 1% formic acid was added. After ultrasonic extraction, the solution was centrifuged at 8000 r/min for 5 min, and the supernatant was transferred to the reagent bottle. Separately, 2 mL of the supernatant was taken, and 100 mg of nine different adsorbent materials were added and vortex-mixed for 30 s, ultrasonicated for 15 min, and centrifuged for 5 min. The supernatant was passed through a 0.22 μm filter membrane and detected by UPLC-MS/MS. Meanwhile, 2 mL of the extract, without any sorbent material, was aspirated as a blank reference. As shown in Figure 3, C_18_ and SAX had substrate purification effects, as observed by examining the adsorption and purification effects of the nine different adsorbents on the feather extract. C_18_ is mainly based on the interaction of nonpolar carbon bonds, which is helpful for the elution and reverse-phase extraction of samples in nonpolar adsorption process. C_18_ was mainly adsorbed on phospholipids and moderately polar compounds. The sample solution treated with C_18_ was clearer. SAX is a strong anion exchange extraction adsorbed on anionic substances. The quaternary ammonium group is always in a cationic state; thus, it has a strong anion exchange function and selectively retains anionic organic compounds (such as compounds containing carboxyl and phenolic hydroxyl groups). Therefore, C_18_ and SAX were selected as candidate materials for further research.

### 2.4. Selection of Adsorbent Dosage

According to the adsorption characteristics of C_18_ and SAX, various materials were added in the same proportion to carry out purification research. However, the results showed that the recovery rate of the related drugs was decreased. By adding C_18_ and SAX in different proportions, that is, when the ratio of SAX to C_18_ was 2:1, the extracted solution was clarified after adsorption purification, impurities were effectively adsorbed, and the matrix effect of the target compound was greatly reduced. Finally, the two solid adsorbents were mixed, and the mixing ratio was set to 2:1, according to the experiment.

At the same time, the amount of adsorbent used was investigated, and the effects of different solid adsorbent dosages (5, 10, 20, 50, 80, and 100 mg) on the purification effect of impurities in 0.1 g feather extract were compared. The results showed that the purification effect of the impurities in the sample was not remarkably improved by continuously increasing the amount of adsorbent when the amount of solid adsorbent reached 50–80 mg. Therefore, the amount of adsorbent added was set to 50 mg. When the adsorbent was added for adsorption and purification, the interference substance in the matrix was effectively removed from the chromatographic peak of the substance to be detected, and the damage of impurities to the instrument and chromatographic column was reduced.

### 2.5. Matrix Effect

Matrix effects should be considered because they are commonly encountered in complex sample matrix LC-MS/MS analyses. The main component of poultry feathers is protein. In this experiment, the MRM mode of mass spectrometry was used for monitoring, which effectively avoided the interference of impurity ions; at the same time, the internal standard quantification method was used. Therefore, the matrix effect was negligible.

The matrix effect was investigated by comparing the results for the QN standard mixture (100 μg/L) dissolved in a 0.1% formic acid solution in water/methanol (90:10, *v*/*v*) and the extracted solution of the noncontaminated duck and chicken samples from different origins and/or different regions. The matrix effect was detected for QNs in duck and chicken feathers (Supplementary Table S1). A total of 10 drugs exhibited a weak signal suppression of 10–20%, and the signal suppressions of other drugs were lower than 10%. The duck feathers had a higher ion suppression activity than the chicken feathers. Therefore, due to sample type variability, the content of QNs in feather samples was determined by the IS method, though not all isotopically labeled ISs for each QNs were attained.

### 2.6. Methodological Validation

The 26 QNs in the feathers were quantified in the method using endogenous markers, and the method was validated for its specificity, linearity, recovery and precision. Selectivity was analyzed and evaluated by 20 blank feathers, and no interferences from other components were observed near the retention time range of QNs in these feathers [41]. Appropriate amounts of mixed standard solutions with seven different concentration points for each QN were added to prepare a standard series of solutions with mass concentrations ranging from 0.5 to 200 μg/L and containing 10 ng/mL of the endogenous marker. Under the selected chromatographic and tandem mass spectrometry conditions, the mass concentration (C, μg/L) of each analyte in the solution was linearly regressed with the MRM peak area A of each analyte. The typical linear regression equation and correlation coefficient (γ) are shown in Table 2, which revealed that the 26 target compounds had good linear relationships within the linear range and the correlation coefficient was not less than 0.992. Ten blank matrix samples of different groups were weighed and determined after being processed through the extraction methods. The detection limit (LOD) was determined according to the signal-to-noise ratio (S/N) of the MRM chromatographic peak, which was greater than 3, and the minimum quantitative limit (LOQ) was determined according to the S/N, which was greater than 10 times. The LOD and LOQ of the 26 target components were 0.12–1.31 and 0.96–2.60 μg/kg, respectively. Twenty-six types of of quinolones in mixed standard solutions with different concentration levels (low, medium, and high) were added to blank chicken feather and duck feather samples to prepare quality control samples with quinolone contents of 10, 100, and 200 μg/kg, respectively. Six parallel determinations were performed for each concentration level and the three replicate batches, with an average recovery rate of the chicken feather samples of 78.9–103% and an intraday and interday reliability less than 10.1 and 13.7; for the duck feather samples, the average recovery rate was 80.9–110%, and the intraday and interday reliability were less than 10.8 and 12.8. The determination results are shown in Table 3.

### 2.7. Quinolone Residue Accumulation in Laying Hens

**Figure 4:**
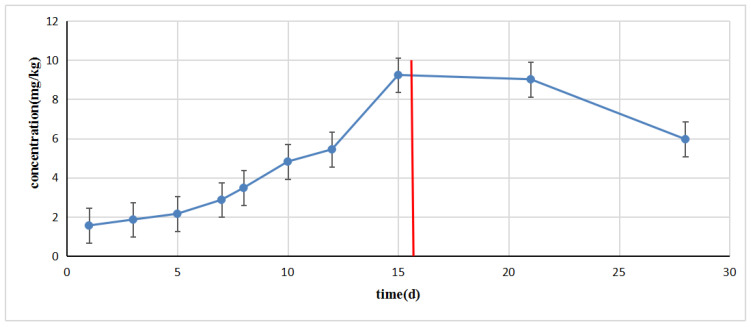
Analysis of feather samples collected in animal experiments. According to the time–drug concentration formation curve, drug accumulation in poultry feathers is a gradual process, demonstrating a smaller accumulation during the initial dosing period, and can be relatively stable during the initial phase of the rest period. Residual elimination is relatively slow. Accumulation in hen feathers after Enrofloxacin feeding.

Hens fed with enrofloxacin were collected at different withdrawal periods. As shown in Figure 4, the residual amount of enrofloxacin increased continuously with the accumulation time. This result indicates that enrofloxacin enters the body, metabolizes, and migrates into feathers in which the empty tubular structure provides a stable place for existence in which drugs have a relatively slow elimination speed and can remain relatively stable for at least 5 days. This mechanism has a great relationship with the pharmacokinetics of quinolones. A quinolone enters the poultry body and is widely distributed in the poultry body through metabolic pathways with a low affinity for plasma proteins and a high affinity for lipids. These characteristics cause quinolones to accumulate in poultry feathers continuously. The hair follicles of feathers are distributed under the skin. Poultry will undergo changes, such as feather metamorphosis, when they grow from a nestling bird to an adult, but the feathers will be fixed and the feather handle will begin to harden. In contrast, the number of hair follicles, which is fixed during the whole development process and is formed from the embryonic development process, is also an important means for drugs to enter feathers. During the whole process of poultry raising, the floor was lifted with grid-like material so that the bottom left the ground, and chicken manure was cleaned regularly. Feather samples were cleaned, wiped, and dried after collection. Therefore, quinolones can be excluded as a possible cause of external contamination from chicken feces.

### 2.8. Application

In this method, total of 50 poultry feather samples were investigated separately for the possible presence of 26 QN drug residues. Two of the chicken feather samples contained ciprofloxacin at 17.13 mg/kg and 12.34 mg/kg, respectively. Eighteen duck feather samples did not contain quinolones. The detection results showed that the quinolone residues in poultry feathers were mainly third-generation quinolone residues, which may be related to the relative stability of the drug ciprofloxacin. In addition, with the rapid development of the scale of livestock farming and the increase in the urban population, the presence of antibiotics in the soil and bodies of water through metabolism poses a serious threat to the health of organisms. Conventional environmental testing relying on machinery, etc., can also serve the purpose. However, compared to biological monitoring, such as through feathers, it lacks bioavailability, and the cumulative effect of antibiotic drugs in organisms cannot be obtained from monitoring data. Changes that are difficult to monitor and difficult to detect can often be more easily monitored using biological means, such as poultry feathers, than physical and chemical means. For poultry, feathers are an important indicator of antibiotic use residues as well as contamination, and feathers have significant advantages over other tissues. The use of feathers meets the principles of conservation of living things, responds to environmentalism, and uses a non-invasive biological material. The feathers are easy to collect and store and can be collected from the same birds without affecting their health. The dangers of antibiotic residues in soil and water cannot be ignored, and conducting these biological studies will allow for a more visual recognition of the threat and a better application of poultry feather monitoring.

## 3. Experimental Section

### 3.1. Chemicals and Materials

A Waters Acquity UPLC–MS/MS instrument (US Waters Company, New Port Richey, FL, USA), intelligent sample grinder (Hede Technology Co., Ltd., Beijing, China), ultrasonic cleaner (Fungilab Company, Barcelona, Spain), high-speed desktop centrifuge (Sigma Company, Louis, MO, USA), QL-901 automatic vortex mixer (Haimen Qilinbeier Instrument Manufacturing Co., Ltd., Nantong, China), and ultrapure water device (purchased from Millipore China, Ltd., Hong Kong, China) were used in the experiment. Chromatographic-grade formic acid, methanol, and acetonitrile were purchased from Fisher Scientific (Waltham, MA, USA). A hydrophilic–lipophilic balance (60 mg, 3 mL), solid-phase extraction column was purchased from Waters. Sodium hydroxide (chemically pure), hydrochloric acid (chemically pure), purified Mili-Q ultrapure water for the experimental water (>18 mω), protease, the 23 mixed standards of quinolone (purity > 98%), balofloxacin, trovafloxacin, and gatifloxacin were purchased from Tianjin Alta Technology Co., Ltd. (Tianjin, China). Twelve QNs of mixed internal standards were purchased from ANPEL Laboratory Technologies (purity > 99%, Shanghai, China); C_8_ (octane bonded to silica gel), SAX (quaternary ammonium halide bonded to silica gel), and SCX (sodium sulfonate bonded to silica gel) were purchased from Agilent Technologies. C_18_ (octadecyl bonded to silica gel), PSA (silica gel-bonded N-propyl ethylenediamine), Florisil (a magnesium oxide composite silica gel adsorbent), Carb (graphite carbon), and Nonocarb (activated carbon nanotubes) were purchased from Agela Technologies. Alumina was purchased from China National Pharmaceutical Group Chemical Reagent Co., Ltd. (Shanghai, China). The multi-adsorption purification filler was made by weighing and evenly mixing 10 g of SAX and 5 g of C_18_ in a triangular flask for later use.

### 3.2. Standard Solution Preparation

Balofloxacin, trovafloxacin, and gatifloxacin standards were accurately weighed, dissolved in methanol, and fixed in volume to prepare a standard stock solution with a mass concentration of 500 mg/L. Twenty-three types of quinolone mixed standards were dissolved in methanol to prepare 1000 mg/L mixed standard stock solution, which was sealed and stored at −20 °C for an effective storage period of not more than six months. A certain amount of each standard stock solution was accurately removed, and 10 μg/mL of mixed standard solution for the 26 quinolone antibiotics was prepared for later use.

### 3.3. Animal Treatment

Forty-eight Hy-Line brown breed chickens (mean weight: 2.25 ± 0.62 kg) were selected for the experiment, and their health status was examined before the experiment. These chickens were randomly divided into groups A and B. Thirty-four chickens were assigned to group A and 14 to group B. The chickens were kept in cages with a grid-like floor at the bottom to effectively avoid contamination by chicken feces. Group A was provided 100 mg/kg^−1^ body weight of 10% enrofloxacin via mixed feeding for 5 days, while group B was the control group without any enrofloxacin addition.

### 3.4. Sample Collection

To monitor residue and its pattern, three chickens were randomly selected from the above experiments, and feathers were collected on 5 consecutive days during the dosing period and on days 1, 3, 5, 7, 8, 10, 12, 14, 21, and 28 during the withdrawal period. Using forceps, the feathers were collected from the roots, placed in sample bags, numbered, and stored at 2–8 °C until further analysis. At the same time, feather samples from 18 ducks and 32 chickens were collected from different sources, such as markets, farms, and slaughterhouses, to avoid contamination. These samples were stored in sealed sample bags at 2–8 °C for analysis and for the verification of methods and drug residue detection.

### 3.5. Sample Pretreatment

After the feather samples were collected, the surfaces were wiped with ultra-pure water to remove dust, feces, and other contaminants, and the water was evaporated and removed in a constant-temperature drying oven at 50 °C. The feathers were cut to a length of approximately 1 cm with scissors and subsequently ground into powder using a sample shaker and grinder. The powder was collected and numbered in sealed sample bags and stored at 2–8 °C under light-proof conditions.

A total of 100 mg of feather sample was weighed (accurate to 0.01 g). To a 10 mL polypropylene centrifuge tube, a certain amount of mixed internal standard stock solution was added, vortexed, and mixed to make it 10 ng/g relative to the content in the tissue. Then, 5 mL of a 1% formic acid and acetonitrile solution was added, vortexed, mixed for 5 min, and left for 30 min. It was then placed on a shaker at 125 r/min and shaken for 1 h at a constant temperature of 40 °C, followed by ultrasonic extraction for 40 min and then centrifugation at 9000 r/min for 5 min. Of the supernatant, 5 mL was pipetted into a 10 mL centrifuge tube, and 50 mg of multiple mechanism adsorption packing was added. It was then vortex mixed and sonicated for 15 min and centrifuged at 9000 r/min for 5 min. The entire supernatant was then pipetted and blown dry under nitrogen at 40 °C. Next, 0.5 mL of a 0.1% formic acid–acetonitrile (9:1, *v*/*v*) solution was added and vortex shaken for 30 s to re-dissolve. This was filtered through a 0.22 μm polytetrafluoroethylene (PTFE) membrane and then measured by UPLC–MS/MS. The sample treatment method for the control group was the same.

### 3.6. Instrumental Analysis

An Acquity UPLC system equipped with a TQS mass spectrometer (Waters Corp., Manchester, UK) was used for the analysis of 26 QNs, while a Waters ACQUITY UPLC BEH C_18_ (inner diameter: 150 × 3.0 mm; particle size: 1.7 μm) was used for chromatographic separation of the 26 QNs. The mobile phase consisted of solvents A (0.1% formic acid in water) and B (0.1% formic acid in ACN). The mobile phase flow rate was 0.3 mL/min for 13 min of total run time, with a linear gradient under the following conditions: 0−6 min, 70% A; 6−9 min, 70−30% A; 9−11 min, 30−20% A; 11−12 min, 20% A; 12−12.11 min, 20−95% A; 12.11–13 min, 95% A.

The detection of the target analytes was carried out on a tandem MS platform with electrospray ionization in the positive ion mode (ESI^+^); the capillary voltage, source temperature, desolvation temperature, cone gas (N_2_) flow rate, and desolvation gas (N_2_) flow rate were set at 2.5 kV, 150 °C, 480 °C, 10 L/h, and 500 L/h, respectively. Data were collected via subsection collection, and the collection time for each analyte was about ±1 min of the peak time. The retention time and multiple reaction monitoring (MRM) conditions are shown in Table 4. The data were acquired and processed using MassLynx 4.1 software.

### 3.7. Parameters for Validation of the Analytical Methodology

The analytical method was validated in the study in terms of its specificity, linearity, recovery, and precision. Twenty feather samples without any substrate were assayed according to the determined method, with 3- and 10-fold signal-to-noise ratios as the limit of detection (LOD) and limit of quantification (LOQ). The recoveries of all compounds were determined by adding standard solutions to uncontaminated feather samples. The precision of the assay was determined by repeated measurements within and between different batches of the samples to which the standard solution was added.

## 4. Conclusions

This study showed that quinolones stably adhered in poultry feathers for a long time, and their accumulation was much higher than the LOD. The proposed UPLC–MS/MS method has the advantages of simplicity, rapidity, environmental protection, a stable recovery rate, and good precision for the simultaneous determination of 26 kinds of quinolone residues in poultry feathers. This method is a new and powerful technique that can play a role in the early warning and supervision of the illegal addition of quinolones in poultry breeding and in monitoring the feather powder that indirectly enters the food chain.

## Figures and Tables

**Table 1:** The table shows the names of nine different adsorption fillers and the types of materials.

Names and Types of Adsorption Materials	
Name	Requirement for Material Type
ALUMINA	Aluminum oxide
SCX	Sodium sulfonate bonded to silica gel
C18	Octadecyl is bonded to silica gel, and the bond end has been treated.
PSA	Silica gel bonded N-propyl ethylenediamine
NONOCARB	activated carbon and zeolite
FLORISIL	Magnesium oxide composite silica gel adsorbent
PESTICARB	Graphite carbon
SAX	Quaternary ammonium halide bonded to silica gel
CARBON NANOTUBE	Multi-walled carbon nanotubes

**Table 2:** The table shows the correlation linearity of 26 QNs with R2 ≥ 0.992 in the range of 0.5–200 ng/mL.The LOD and LOQ of 26 target components were, respectively, 0.12–1.31 and 0.96–2.60 μg/kg.

Standard Curve, Correlation Coefficient, Detection Limit, and Quantitative Limit of 26 QNs					
Drug	Linear Equation	R2	Concentration Range (ng/mL)	LOD (μg/kg)	LOQ (μg/kg)
NAL	y = 2927.6 (±0.05) x − 8767.8 (±1.07)	0.994	0.5–50	0.16	1.08
OXO	y = 37.002 (±0.025) x − 166.02 (±0.31)	0.997	0.5–50	0.54	1.8
NOR	y = 28.576 (±0.019) x + 251.17 (±0.25)	0.992	1–100	0.27	1.23
LOM	y = 191.87 (±0.008) x + 330.79 (±0.03)	0.993	1–100	0.17	1.32
DAN	y = 39.122 (±0.008) x − 98.238 (±0.04)	0.999	2–200	0.68	2.20
NAD	y = 1126.9 (±0.52) x − 1311.2 (±1.15)	0.997	1–100	0.22	1.15
OFX	y = 24.466 (±0.005) x + 38.5 (±0.03)	0.995	1–100	0.56	1.87
GAT	y = 17.266 (±0.004) x + 274.96 (±0.28)	0.996	2–200	1.31	2.01
GEM	y = 14.482 (±0.03) x + 44.762 (±0.10)	0.998	2–200	1.20	2.60
SPA	y = 119.28 (±0.02) x + 58.265 (±0.39)	0.999	0.5–50	0.19	0.96
MOX	y = 46.803 (±0.02) x − 189.76 (±0.28)	0.995	1–100	0.34	1.14
TOS	y = 168.78 (±0.17) x + 320.1 (±0.43)	0.999	1–100	0.15	1.36
FLU	y = 16.278 (±0.02) x + 54.404 (±0.15)	0.994	2–200	0.68	2.2
CIN	y = 236.96 (±0.12) x − 514.08 (±0.49)	0.996	0.5–50	0.40	0.98
PPA	y = 12.445 (±0.02) x + 79.361 (±0.23)	0.995	1–100	0.68	1.73
ENO	y = 60.418 (±0.007) x − 40.661 (±0.06)	0.998	1–100	0.83	1.70
CIP	y = 434.24 (±0.029) x + 6606.7 (±0.08)	0.998	1–100	0.12	1.14
BAL	y = 148.93 (±0.045) x − 271.78 (±0.65)	0.994	1–100	0.13	1.44
ENR	y = 154.65 (±0.023) x + 3905.2 (±0.41)	0.993	2–200	0.30	1.99
MAR	y = 54.164 (±0.005) x + 37.6 (±0.40)	0.997	2–200	0.20	1.78
FLE	y = 1069.7 (±0.058) x + 3312 (±0.41)	0.997	1–100	0.27	1.12
SAR	y = 541.99 (±0.027) x + 246.77 (±0.17)	0.998	1–100	0.31	1.78
ORB	y = 200.56 (±0.011) x − 577.78 (±0.05)	0.995	2–200	0.58	2.32
DIF	y = 515.72 (±0.008) x − 509.96 (±0.08)	0.996	2–200	1.15	2.19
TRO	y = 16.459 (±0.003) x + 33.812 (±0.09)	0.994	2–200	0.75	2.50
GAR	y = 111.08 (±0.088) x + 46.782 (±0.32)	0.995	1–100	0.19	1.64

y represents the peak area of a certain concentration of mixed standard solution analyzed by the instrument under certain conditions; x represents the concentration of the mixed standard solution to be measured.

**Table 3:** The feather samples of chickens and ducks without any matrix were selected and added with appropriate amounts of the mixed standard substances so that the concentrations of various compounds contained in the samples were 10, 100, and 200 ng/g, respectively. The determination was repeated six times, and the batches were repeated three times using the above method. The recoveries of their 26 compounds at different spiked concentrations were as follows.

Recovery and Precision of the Method for 26 QNs in Poultry Feathers							
Drugs	Add Value	Recovery (%)		Intraday Precision (RSD, %) (*n* = 6, 3 Days)		Interday Precision (RSD, %) (*n* = 18, 3 Days)	
		Chicken	Duck	Chicken	Duck	Chicken	Duck
NAL	10	97.8	89.6	5.0	5.8	10.5	2.4
	100	99.6	97.6	6.2	8.9	9.4	12.7
	200	102	102	6.2	4.8	6.8	10.1
OXO	10	79.2	82.0	7.3	1.3	11.3	2.4
	100	88.3	80.8	1.6	3.0	5.8	3.1
	200	90.1	107	7.6	7.5	2.7	5.7
NOR	10	83.3	89.7	4.5	2.9	7.9	9.9
	100	91.2	91.8	8.8	3.5	4.2	7.9
	200	99.7	98.1	6.8	6.0	8.6	11.0
LOM	10	78.9	93.7	9.7	8.4	5.9	3.4
	100	89.6	105	8.7	8.4	6.8	11.2
	200	94.8	107	6.9	6.9	5.4	4.6
DAN	10	96.7	90	2.9	6.8	9.9	6.1
	100	98.2	95.4	1.3	9.5	13.2	7.5
	200	101	95.1	1.8	2.0	10.3	12.1
NAD	10	87.1	81.1	5.4	1.6	8.8	10.2
	100	103	94.2	6.2	10.8	7.6	3.3
	200	102	110	8.4	5.2	6.6	4.5
OFX	10	92.3	89.7	9.3	3.7	9.4	6.6
	100	97.6	99.5	8.9	8.2	11.3	6.7
	200	96.1	98.4	6.7	7.6	6.1	7.2
GAT	10	90.9	91.0	7.5	1.11	8.6	1.6
	100	96.2	101	1.68	1.85	7.1	12.1
	200	97.7	104	1.9	7.5	9.2	4.5
GEM	10	89.1	87.5	1.6	0.6	7.7	7.6
	100	93.2	101	0.8	5.6	11.9	9.3
	200	98.6	99.4	2.3	3.9	5.3	10.0
SPA	10	87.6	92.7	4.3	9.8	4.4	8.5
	100	90.8	95.1	2.6	4.8	3.7	4.9
	200	91	94.9	1.7	1.1	5.2	6.7
MOX	10	97.0	86.8	3.8	0.45	1.7	5.3
	100	98.9	91.3	8.2	2.96	9.5	3.7
	200	99.6	99.2	0.22	1.9	4.2	12.8
TOS	10	86.4	89.9	10.1	5.7	7.0	4.9
	100	88.8	92	5.9	4.3	1.1	6.7
	200	90.1	87.5	3.8	3.7	3.6	2.2
FLU	10	97.3	99.4	0.15	10.2	12.6	3.1
	100	103	103	8.4	0.6	13.7	7.9
	200	98.5	97.1	0.4	4.9	7.9	4.5
CIN	10	91.8	92.3	1.9	2.8	2.6	5.1
	100	92.4	96.8	6.4	7.5	5.6	7.3
	200	97.6	89.8	7.1	1.3	8.9	12.4
PPA	10	88.4	90	1.7	5.2	9.8	3.9
	100	89.7	93.3	8.6	8.4	12.4	9.3
	200	92.6	91.2	0.8	5.4	6.1	4.6
ENO	10	87.1	89.7	6.5	1.9	12	7.2
	100	90.2	93.2	4.2	2.3	1.1	1.8
	200	98.3	85.1	8.8	2.9	5.0	2.0
CIP	10	92.7	93.8	3.8	0.1	14.6	3.7
	100	95.6	99.8	9.8	1.2	7.8	8.6
	200	94.6	95.9	7.1	8.9	8.9	9.4
BAL	10	88.7	99.5	8.8	4.9	8.0	10.1
	100	96.6	103	3.7	2.7	2.5	3.6
	200	96.2	93.2	8.1	9.1	2.7	6.4
ENR	10	94.5	88.8	3.3	1.7	8.2	1.3
	100	98.3	92.7	9.1	2.1	4.6	7.8
	200	96.9	89.5	7.8	6.2	7.1	8.4
MAR	10	96.7	99.2	2.5	5.7	5.6	4.3
	100	97.5	101	8	1.2	13.3	2.1
	200	98.7	97.9	8.3	3.4	7.8	6.6
FLE	10	95.3	97.3	3.4	3.3	8.1	9.7
	100	98.2	97.6	9.4	5.4	9.4	7.4
	200	97.3	97.7	1.5	8.7	12.6	5.9
SAR	10	100.2	96.7	7.1	2.7	3.7	11.3
	100	97.6	88.3	8.2	3.4	11.9	5.8
	200	102	89.6	8.5	5.9	9.0	9.6
ORB	10	89.9	89.6	2.7	2.1	13.7	8.7
	100	93.7	87.8	7.8	4.9	8.6	6.4
	200	98.1	88.7	6.6	3.7	11.7	5.3
DIF	10	91.3	87.6	5.1	1.7	10.4	5.5
	100	98.2	92.7	1.1	2.4	5.7	6.7
	200	90.8	87.5	8.3	0.6	3.6	11.4
TRO	10	95.7	91.4	5.9	1.4	10.0	9.7
	100	97.2	96.5	9.8	3.3	5.8	10.7
	200	98.9	99.2	3.7	8.9	5.6	3.8
GAR	10	91.3	90.7	1.3	8.7	7.2	2.6
	100	90.7	85.4	6.7	5.5	2.1	5.8
	200	91.4	91.3	2.6	7	3.8	6.9

**Table 4:** The table shows the peak times, qualitative and quantitative ion pairs, collision energies, and cone hole voltages for the 26 QNs and 12 internal standards.

Retention Time and MS Condition of 26 QNs and IS					
Drug	Time (min)	Qualitative ion Pair (*m*/*z*)	Quantitative ion Pair (*m*/*z*)	Cone (V)	CE (eV)
NAL	8.99	233 > 187 233 > 215 ^a^	233 > 215	24	25
OXO	7.38	262 > 160 262 > 215.9 ^a^	262 > 215.9	24	25
NOR	4.17	320.2 > 275 320.2 > 302 ^a^	320.2 > 302	24	25
LOM ^d^	4.36	352.1 > 265.1 352.1 > 308.1 ^a^	352.1 > 308.1	24	25
DAN	4.39	357.9 > 283 357.9 > 340 ^a^	357.9 > 340	30	20 25
NAD ^c^	8.70	361.14 > 283.3 361.14 > 343.2 ^a^	361.14 > 343.2	24	20
OFX	4.20	361.14 > 261.1 361.14 > 318.1 ^a^	361.14 > 320.2	24	25
GAT ^d^	4.99	376.3 > 261.2 376.3 > 358.3 ^a^	376.3 > 358.3	24	25 20
GEM ^d^	5.69	390.4 > 313.3 390.4 > 372.3 ^a^	390.4 > 313.3	24	25 20
SPA ^b^	5.34	393.2 > 292.1 393.2 > 349.1 ^a^	393.2 > 292.1	24	25
MOX	5.75	402.4 > 261.1 402.4 > 384.4 ^a^	402.4 > 384.4	24	25
TOS ^e^	5.91	405.2 > 261.2 405.2 > 387.2 ^a^	405.2 > 387.2	24	25
FLU ^e^	7.37	262 > 160 262 > 201.9 ^a^	262.0 > 201.9	28	46 38
CIN ^e^	6.52	263 > 189.1 263 > 217 ^a^	263 > 217	24	25 20
PPA	3.82	304.1 > 215. 304.1 > 217.4 ^a^	304.1 > 217.5	24	40 30
ENO	4.01	320.9 > 231.1 320.9 > 235.7 ^a^	320.9 > 231.1	24	25 20
CIP	4.28	332.1 > 288 332.1 > 314 ^a^	332.1 > 314	25	20 23
BAL ^d^	4.39	357.9 > 283 357.9 > 340 ^a^	357.9 > 340	30	20 25
ENR	4.63	360.3 > 245 360.3 > 316 ^a^	360.3 > 316	45	30 20
MAR ^c^	4.11	363.3 > 320 363.3 > 345 ^a^	363.3 > 345	32	16 21
FLE ^d^	4.26	369.9 > 235.7 369.9 > 325.9 ^a^	369.9 > 325.9	24	20 25
SAR	5.27	386 > 299 386 > 342 ^a^	386 > 342	30	31 23
ORB ^b^	4.86	396.4 > 295 396.4 > 352 ^a^	396.4 > 352	33	25 18
DIF	5.38	400.2 > 299 400.2 > 382 ^a^	400.2 > 382	30	30
TRO ^e^	6.23	417.1 > 330.1 417.1 > 399.1 ^a^	417.1 > 370.1	26	44 46
GAR ^e^	7.04	427.1 > 286 427.1 > 410 ^a^	427.1 > 366.2	12	44 38
OXO-D5		266.2 > 197.4	266.2 > 247.9	10	20
SAR-D8		393.2 > 303.4	393.2 > 349	10	20
ENO-D8		329.2 > 98.9	329.1 > 174.9	10	20
OFX-D3		365.1 > 260.9	365.1 > 321.2	10	20
CIP-D8		340.2 > 296.1	340.2 > 322.2	10	20
DAN-D3		361.2 > 317.2	361.2 > 342.9	10	20
NOR-D5		325.2 > 238	325.2 > 281.1	10	20
NAL-D5		238.1 > 188.1	238.1 > 220	10	20
PPA-d5		338.16 > 274.1	338.2 > 293.9	10	20 30
ENR-D5		365.2 > 321.1	365.2 > 347.1	10	20 30
DIF-D3		392.5 > 318.4	392.5 > 362.1	10	20
MOX-d3		266.9 > 215	266.9 > 249.1	10	20

^a^ Quantitative ion used for quantification with an isotopically labeled analogue as the IS. ^b^ Product ion used for quantification with DIF-D3 as the IS. ^c^ Product ion used for quantification with OFX-D3 as the IS. ^d^ Quantitative used for quantification with ENR-D5 as the IS. ^e^ Product ion used for quantification with MOX-D3 as the IS.

## Supplementary Materials

The following supporting information can be downloaded at: https://www.mdpi.com/article/10.3390/molecules28093738/s1, Table S1: Matrix effects of 26 quinolones.

## Sample Availability

Samples of the compounds are available from the authors.

## Abbreviations

Quinolones	QNS
Norfloxacin	NOR
Nadifloxacin	NAD
Gemifioxacin Mesylate	GEM
Tosufloxacin Tosilate	TOS
Pipemidic Acid	PPA
Balofloxacin	BAL
Fleroxacin	FLE
Difloxacin	DIF
Nalidixic Acid	NAL
Lomefloxacin	LOM
Ofloxacin	OFX
Sparfloxacin	SPA
Flumequine	FLU
Enoxacin	ENO
Enrofloxacin	ENR
Sarafloxacin	SAR
Trovafloxacin	TRO
Oxolinic Acid	OXO
Danofloxacin	DAN
Gatifloxacin	GAT
Moxifloxacin	MOX
Cinoxacin	CIN
Ciprofloxacin	CIP
Marbofloxacin	MAR
Orbifloxacin	ORB
Garenoxacin	GAR
