# Supplementary material for: Multiresidue Determination of 26 Quinolones in Poultry Feathers Using UPLC-MS/MS and Their Application in Residue Monitoring
Source: Molecules. 2023 Apr 26;28(9):3738. doi: 10.3390/molecules28093738 (PMC10180027; doi:10.3390/molecules28093738)
Supplement: Supplementary file 1 [file molecules-28-03738-s001.zip › molecules-2284870-supplementary.pdf]

Table S1. Matrix effects of 26 quinolones.

Drug	Chicken	Duck	(RSD, %)	(RSD, %)
NAL	4.6	4.9	1.1	2.5
OXO	10.2	12.4	2.3	2.8
NOR	3.8	6.7	0.8	0.9
LOM	5.2	8.1	4.6	5.1
DAN	11.6	15.7	6.1	7.3
NAD	3.0	7.2	3.1	3.7
OFX	1.1	2.8	1.9	2.9
GAT	13.2	14.8	4.8	5.6
GEM	3.2	5.4	2.4	3.7
SPA	12.9	16.4	7.3	8.5
MOX	2.8	3.7	6.6	6.9
TOS	1.9	2.5	1.7	2.4
FLU	6.1	8.3	0.6	1.8
CIN	16.6	19.5	6.7	8.2
PPA	7.3	9.1	4.9	5.5
ENO	12.7	13.1	3.0	3.7
CIP	4.5	6.2	1.7	3.1
BAL	14.9	15.5	8.6	9.4
ENR	4.3	5.7	0.7	0.9
MAR	12.8	13.2	7.8	8.4
FLE	8.0	8.5	3.8	4.2
SAR	14.1	16.5	1.4	3.5
ORB	5.6	5.9	5.9	6.6
DIF	2.7	3.4	3.1	4.0
TRO	4.5	4.9	1.6	2.6
GAR	11.4	14.6	2.4	3.5
